# The pancreatic exocrine function in patients with pancreatic endocrine insufficiency: the evaluation with cine-dynamic magnetic resonance cholangiopancreatography using a spatially selective inversion-recovery pulse and T1 mapping

**DOI:** 10.1007/s11604-022-01256-3

**Published:** 2022-03-02

**Authors:** Mayumi Higashi, Masahiro Tanabe, Teppei Yonezawa, Matakazu Furukawa, Etsushi Iida, Katsuyoshi Ito

**Affiliations:** Department of Radiology, Yamaguchi University Graduate School of Medicine, 1-1-1 Minami-Kogushi, Ube, Yamaguchi 755-8505 Japan

**Keywords:** Pancreas, Cine-dynamic MRCP, MR imaging, Diabetes mellitus

## Abstract

**Purpose:**

To evaluate the association of the pancreatic exocrine function estimated by cine-dynamic magnetic resonance cholangiopancreatography (MRCP) using a spatially selective inversion-recovery (IR) pulse with the pancreatic endocrine function estimated by the T1 relaxation time of the pancreatic parenchyma and HbA1c values.

**Materials and methods:**

Forty-three patients with suspected hepatobiliary or pancreatic diseases were included. Patients were classified into three groups: HbA1c < 5.7% (normal group), 5.7% ≤ HbA1c < 6.5% (prediabetes group), and HbA1c ≥ 6.5% (diabetes group). The frequency of the secretory flow of the pancreatic juice was observed within the area of the IR pulse, and the moving distance (mean secretion grade) of the pancreatic juice inflow within the area of the IR pulse on cine-dynamic MRCP, and the T1 relaxation time of the pancreatic parenchyma on the T1 map images were assessed. The MR imaging measurements were compared using Spearman’s rank correlation coefficient analysis and the Kruskal–Wallis and Mann–Whitney *U* tests.

**Results:**

Both the mean secretion grade and frequency of the pancreatic secretory inflow had a significant negative correlation with the T1 relaxation time of the pancreatic parenchyma (*r* = − 0.335, *p* = 0.028 and *r* = − 0.305, *p* = 0.047, respectively) and HbA1c values (*r* = − 0.308, *p* = 0.044 and *r* = − 0.313, *p* = 0.041, respectively). Both the mean secretion grade and frequency of the pancreatic secretory inflow in the elevated HbA1c (prediabetes and diabetes) group were significantly lower than those in the normal group (*p* = 0.030 and *p* = 0.029, respectively).

**Conclusion:**

The pancreatic exocrine function estimated by cine-dynamic MRCP was significantly lower in patients with prediabetes and diabetes than in controls. Cine-dynamic MRCP with a spatially selective IR pulse may be useful for the early diagnosis of pancreatic exocrine insufficiency in patients with pancreatic endocrine insufficiency.

## Introduction

The endocrine and exocrine tissues of the pancreas reciprocally interact to coordinate the activities of digestion, absorption, and glucose metabolisms [1]. Insulin, as the islet-derived hormone, regulates the exocrine function and mediates the interplay between the endocrine and exocrine pancreas [2]. Therefore, impairment of the pancreatic endocrine function can cause pancreatic exocrine insufficiency.

Diabetes mellitus (DM) is one of the most common endocrine disorders, resulting from insulin resistance and insulin deficiency and accompanied by an impaired exocrine function that manifests as an insufficient activity of digestive enzymes. Pancreatic exocrine insufficiency has been reported to be present in a considerable proportion of patients with DM (26%-74% of type 1 DM patients and 10%-56% of type 2 DM patients) [3]. Patients with pancreatic exocrine insufficiency often have symptoms including steatorrhea, abdominal pain, abdominal bloating and weight loss, mainly due to the maldigestion and malabsorption of fat [4], and these symptoms can significantly affect the quality of life. Therefore, the early and accurate diagnosis of pancreatic exocrine insufficiency is essential for the effective treatment and improvement of the quality of life in DM patients.

To facilitate the early diagnosis of pancreatic exocrine insufficiency, indirect pancreatic function tests, especially the measurement of fecal elastase-1 (FE-1) in spot stool, have been used in clinical practice. FE-1 has demonstrated good sensitivity and specificity in moderate and severe pancreatic exocrine insufficiency (sensitivity, 100%; specificity, 93%); however, in mild cases of pancreatic exocrine insufficiency, the sensitivity is inadequate (sensitivity, 63%) [5].

Magnetic resonance imaging (MRI) have been increasingly used to evaluate the impairment of the pancreatic endocrine and exocrine function in patients with pancreatic diseases, such as DM and chronic pancreatitis (CP) [6–11]. A previous study demonstrated that cine-dynamic MR cholangiopancreatography (MRCP) with a spatially selective inversion-recovery (IR) pulse capable of visualizing the secretory flow of pancreatic juice had potential utility for estimating the pancreatic exocrine function noninvasively [6]. Another study showed that the T1 relaxation time of the pancreatic parenchyma measured by T1 mapping, which may reflect parenchymal fibrotic changes, was significantly increased in patients with an impaired glucose tolerance [11]. However, no previous study has evaluated the pancreatic exocrine function in patients with impaired glucose tolerance and DM using MRI.

Therefore, the present study evaluated the association of the pancreatic exocrine function estimated by cine-dynamic MRCP using a spatially selective IR pulse with the pancreatic endocrine function estimated by the T1 relaxation time of the pancreatic parenchyma and HbA1c values.

## Materials and methods

### Study population

The institutional review board of our hospital approved this retrospective study, and the need for patient informed consent was waived. Using our radiology reporting database system, we searched for patients suspected of having hepatobiliary or pancreatic diseases who underwent abdominal MRI, including T1 mapping and cine-dynamic MRCP with a spatially selective IR pulse, between March 2019 and January 2020.

Among these patients, we identified 49 patients who underwent an HbA1c test within 1 month before or after MRI because of the clinical requirement for the assessment of glucose tolerance. Six of these patients were excluded for the following reasons: incomplete cine-dynamic MRCP (*n* = 1); unclear visualization of the main pancreatic duct (*n* = 2); large tumors of the pancreatic head that hampered the evaluation of the pancreatic juice inflow on cine-dynamic MRCP (*n* = 2); and severe fatty infiltration (*n* = 1). Thus, 43 consecutive patients (26 men, 17 women; mean age, 70.4 ± 11.3 years; range, 26–88 years) were included in our study. These included patients with pancreatic diseases (*n* = 19), such as branch-duct intraductal papillary mucinous neoplasms (*n* = 12, median size 8.5 mm, interquartile range, 7.75–12.75 mm), pancreatic cyst (*n* = 1), pancreatic ductal dilatation with unknown causes (n = 4) and chronic pancreatitis (*n* = 2); patients with hepatobiliary diseases (*n* = 22) such as acute cholecystitis (*n* = 4), biliary stones (*n* = 5), cholangitis (*n* = 3), adenomyomatosis (*n* = 2), choledochal cyst (*n* = 1), peribiliary cyst (*n* = 1), intrahepatic bile ductal dilatation with unknown cause (*n* = 1), gallbladder polyp (*n* = 1), gallbladder cancer (*n* = 1), hepatocellular carcinoma (*n* = 1), hepatic hemangioma (*n* = 1) and fatty liver (*n* = 1); and patients with no diseases (*n* = 2). The patients were classified into three groups based on the American Diabetes Association criteria using HbA1c values as follows: normal group, HbA1c < 5.7% (*n* = 12); prediabetes group, 5.7% ≤ HbA1c < 6.5% (*n* = 15); and diabetes group, HbA1c ≥ 6.5% (*n* = 16).

### MRI technique

**Fig. 1:**
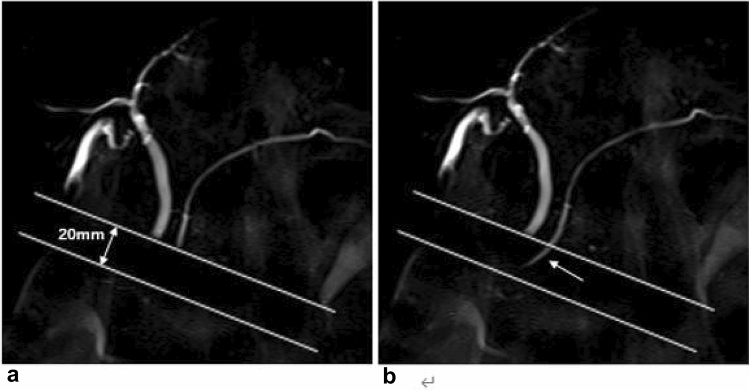
Imaging findings of cine dynamic MRCP with a spatially selective IR pulse. **a** A spatially selective IR pulse (inversion time, 2200 ms) with a width of 20 mm was placed on the pancreatic head perpendicular to the main pancreatic duct to null the static pancreatic juice signal. The static pancreatic juice within the area of the spatially selective IR pulse was shown as low signal intensity. **b** The secretory flow of the pancreatic juice was observed as high signal intensity (arrow) within the area of the spatially selective IR pulse. The secretion grade of the pancreatic juice was categorized as grade 3. MRCP with a spatially selective IR pulse was repeatedly performed every 15 s (5 s of scanning and 10 s of rest) during 5 min (total of 20 images), and a series of these MRCP images was defined as cine-dynamic MRCP with a spatially selective IR pulse

MRI was performed with a 3.0-T MR system (Vantage Galan ZGO; Canon Medical Systems, Tochigi, Japan) equipped with a 16-channel body coil. Patients were required to fast for at least 4 h before MR examinations. At the MR examination, 36 mg of manganese chloride tetrahydrate (Bothdel Oral Solution, 250 mL; Kyowa Hakko Kirin, Tokyo, Japan) was ingested orally to reduce the signal from the bowel. Cine-dynamic MRCP with a spatially selective IR pulse and T1 mapping were routinely performed as part of our hepatobiliary and pancreatic MR examination protocol. First, a breath-hold, thick-slab 2D MRCP was obtained as a reference image to depict the main pancreatic duct in the coronal plane. The imaging parameters were as follows: repetition time (TR)/echo time (TE), 5000/507 ms; echo train spacing, 6.5 ms; slice thickness, 50 mm; acquisition matrix, 512 × 512; field of view (FOV), 350 × 350 mm; and bandwidth, 558 Hz/pixel. Then, using the same MR sequence, a spatially selective IR pulse (inversion time, 2200 ms) with a width of 20 mm was placed on the pancreatic head perpendicular to the main pancreatic duct to null the static pancreatic juice signal. With this method, the static pancreatic juice in the areas subjected to a spatially selective IR pulse appeared dark, while the inflow of the pancreatic juice in these areas was observed as a high signal intensity. MRCP with a spatially selective IR pulse was repeatedly performed every 15 s (5 s of scanning and 10 s of rest) during 5 min (total of 20 images), and a series of these MRCP images was defined as cine-dynamic MRCP with a spatially selective IR pulse (Fig. 1).

T1 maps were acquired using the modified Look-Locker method. The following imaging parameters were used: TR/TE, 3.4/1.3 ms; flip angle, 13°; slice number,1 slice; slice thickness, 8 mm; acquisition matrix, 192 × 256; FOV, 280 × 360 mm; parallel imaging factor, 2; and band width, 781 Hz/pixel.

### Image analyses

**Fig. 2:**
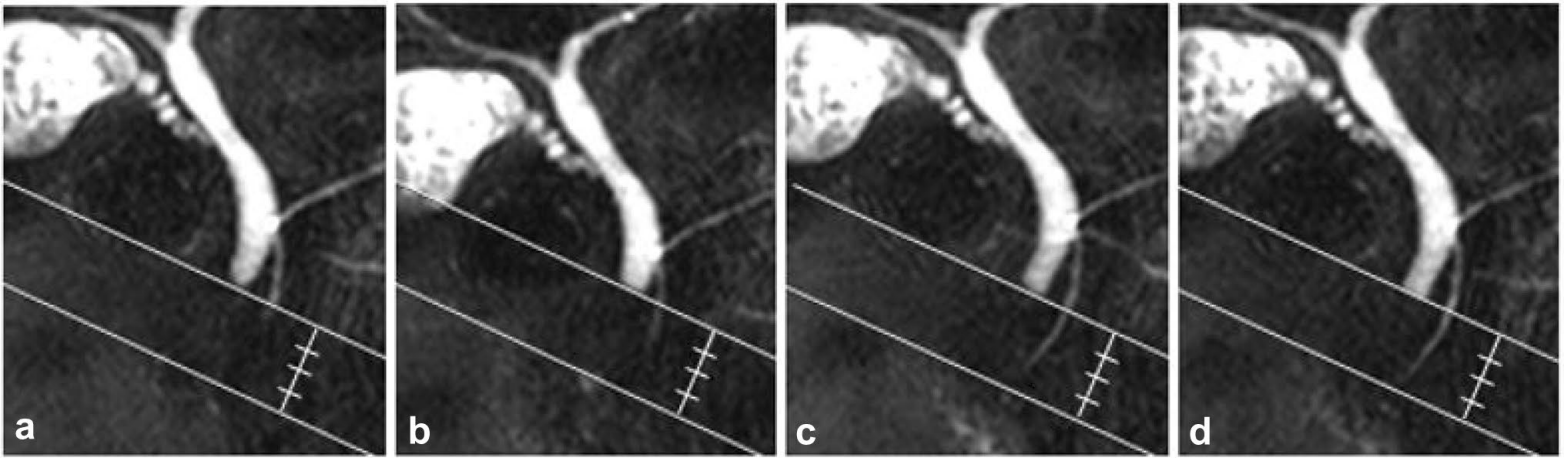
Categorization of the secretion grade based on the distance that the pancreatic juice flowed within the area of the spatially selective IR pulse. **a** Grade 1 (less than 5 mm), **b** grade 2 (5–10 mm), **c** grade 3 (11–15 mm), **d** grade 4 (more than 15 mm)

**Fig. 3:**
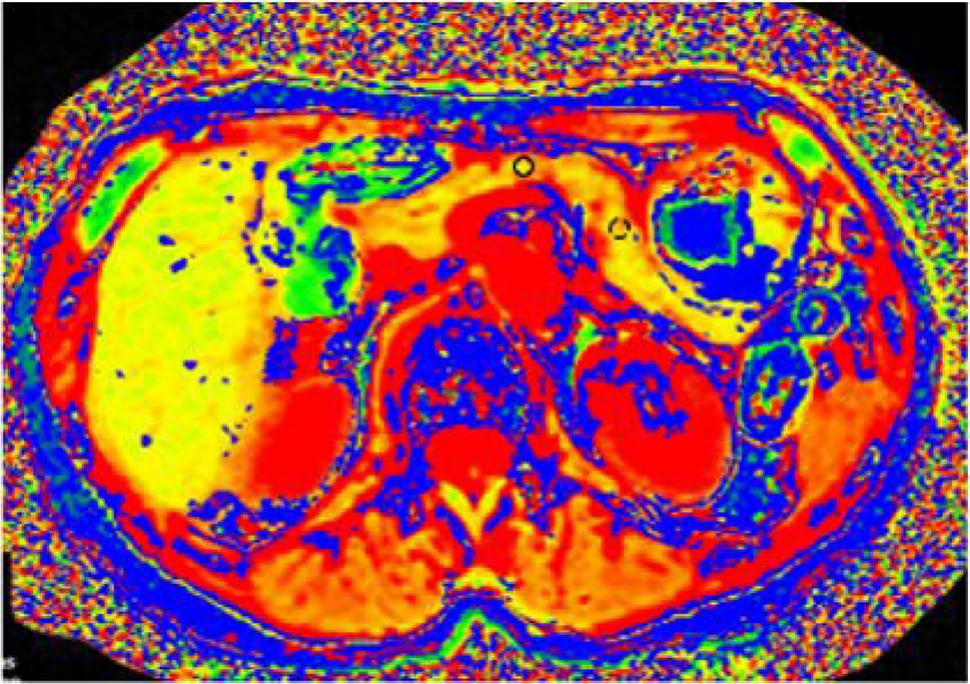
T1 map image in an 81-year-old woman with prediabetes. The T1 relaxation times of body (circle ROI) and tail (dashed circle ROI) of the pancreas were 889 ms and 978 ms, respectively. The mean T1 relaxation time was 934 ms

Cine-dynamic MRCP images were evaluated independently by 2 radiologists (M.H. and K.I., with 5 and 32 years of clinical experience, respectively) who were blinded to any clinical information of the patients, and any interpretation discrepancies were resolved by reaching a consensus. MR images were evaluated for (a) the frequency of the pancreatic juice secretion, defined as the frequency of the detection of a high signal inflow of the pancreatic juice within the area with a spatially selective IR pulse, and (b) the secretion grade of the pancreatic juice, which was assigned according to the distance that the pancreatic juice flowed into the area of a spatially selective IR pulse using the 5-point secretion grading score (grade 0, no secretion; grade 1, < 5 mm; grade 2, 5-10 mm; grade 3, 11-15 mm; grade 4, > 15 mm) (Fig. 2). The mean secretion grade was defined as follows: (total grading score)/20. In addition, the reviewers measured the T1 relaxation time of the pancreatic parenchyma on the T1 map images using operator-defined regions of interest (ROIs), in consensus. Effort was made to draw the ROI circles as large as possible in a homogeneous area of the pancreatic parenchyma while avoiding the dilated pancreatic duct, retroperitoneal fat and artifacts. The ROI measurements were made at two places on the body or tail of the pancreas, and the average values were used for the analysis (Fig. 3).

### Statistical analyses

Statistical analyses were performed using the SPSS software program for Windows, version 18.0 (IBM Corp., Armonk, NY, USA). A Spearman’s rank correlation coefficient analysis was performed to evaluate the correlation between the MRI measurements and HbA1c values. Kruskal–Wallis and Mann–Whitney *U* tests were conducted to compare the MRI measurements among the groups. A *p* value less than 0.05 was considered to indicate a significant difference. The interobserver agreement between the two radiologists was also evaluated using Cohen κ statistics. We interpreted the κ values as follows: ≤ 0.40, poor agreement; 0.41–0.60, moderate agreement; 0.61–0.80, substantial agreement; and ≥ 0.81, excellent agreement.

## Results

**Fig. 4:**
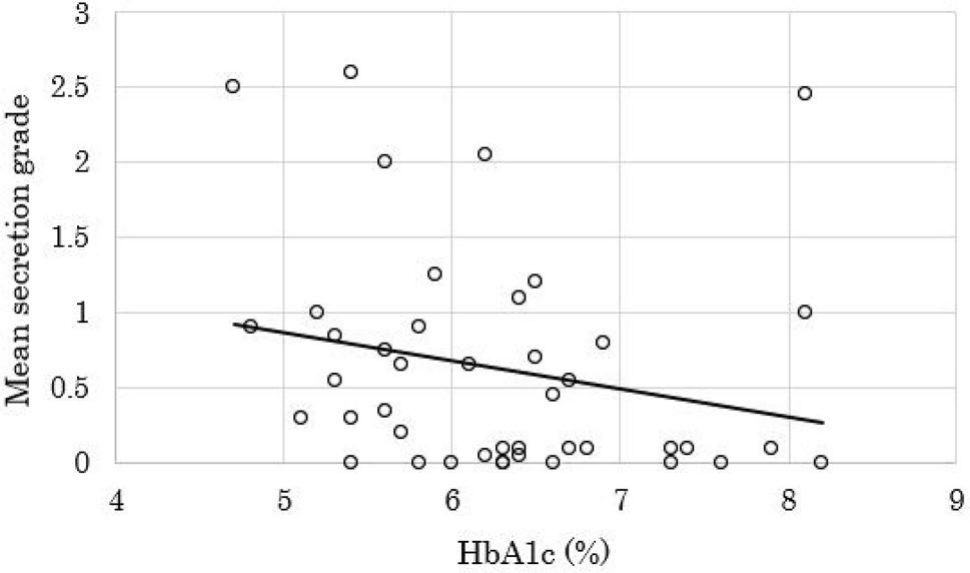
Correlation between the mean secretion grade and HbA1c value. There was a significant negative correlation between the mean secretion grade and HbA1c value (*r* = − 0.308, *p* = 0.044)

**Fig. 5:**
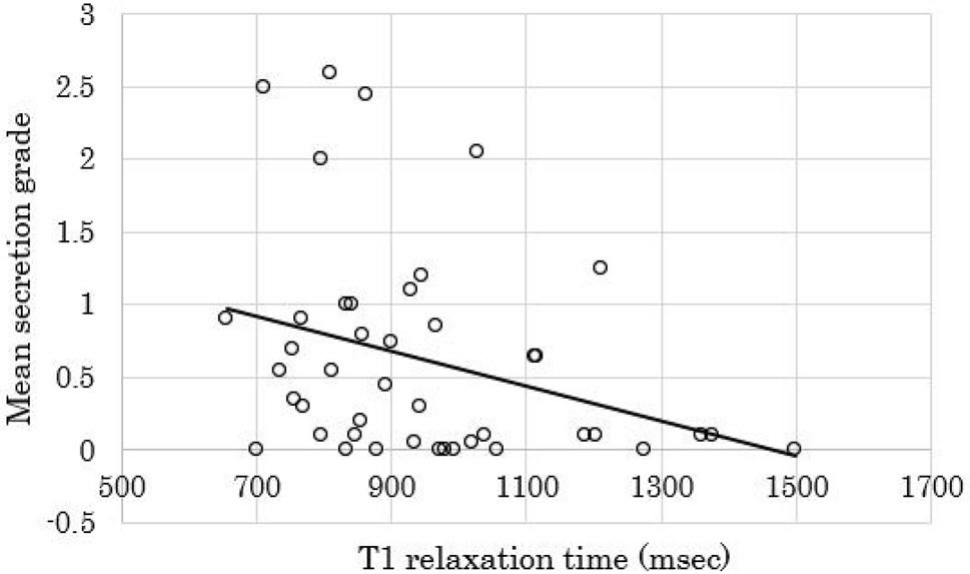
Correlation between the mean secretion grade and T1 relaxation time of the pancreatic parenchyma. There was a significant negative correlation between the mean secretion grade and T1 relaxation time of the pancreatic parenchyma (*r* = − 0.335, *p* = 0.028)

In the HbA1c test, the mean HbA1c value was 6.3% ± 0.9% (range 4.7%-8.2%) in all patients. The median T1 relaxation times of the pancreatic parenchyma on the T1 map images was 899 ms (range 656–1498 ms; interquartile range 811–1033 ms). In cine-dynamic MRCP with a spatially selective IR pulse, the median frequency of the pancreatic juice inflow in all patients was 7 times (range 0–20 times; interquartile range 1–14.5 times) in a series of 20 images. The median mean secretion grade of the pancreatic juice in all patients was 0.35 (range 0–2.6; interquartile range 0.075–0.9). The interobserver agreement for the detection of the inflow of the pancreatic juice and the grading of the moving distance of the pancreatic juice with cine-dynamic MRCP was excellent (κ value = 0.927). Regarding the relationship of the HbA1c values with the mean secretion grade and frequency of the pancreatic secretory inflow, the HbA1c values had significant negative correlations with both the mean secretion grade (*r* = − 0.308, *p* = 0.044) (Fig. 4) and the frequency of the pancreatic secretory inflow (*r* = − 0.313, *p* = 0.041). In contrast, the HbA1c values had a significant positive correlation with the T1 relaxation time of the pancreatic parenchyma (*r* = 0.408, *p* = 0.007). In addition, the T1 relaxation time of the pancreatic parenchyma had significant negative correlations with both the mean secretion grade (*r* = − 0.335, *p* = 0.028) (Fig. 5) and the frequency of the pancreatic secretory inflow (*r* = − 0.305, *p* = 0.047).

In the comparison among the 3 groups (Table 1), the T1 relaxation time of the pancreatic parenchyma in the normal group (median, 803 [interquartile range 750–910] msec) was significantly shorter than that in the prediabetes (median, 934 [interquartile range 850–1024] msec) and diabetes groups (median, 968 [interquartile range 851–1191] msec) (*p* = 0.034). In cine-dynamic MRCP findings, no significant differences were noted in the mean secretion grade (*p* = 0.095) and frequency of the pancreatic secretory inflow (*p* = 0.093) among the 3 groups. However, there were trends toward significances in both the mean secretion grade (median, 0.8 [interquartile range 0.34–1.25] vs. median, 0.1 [interquartile range 0.025–0.775], *p* = 0.066) and the frequency of the pancreatic secretory inflow (median, 12 [interquartile range 6.75–16] times vs. median, 2 [interquartile range 0.5–13.5] times, *p* = 0.059) between the normal group and the prediabetes group although the differences were not statistically significant. Comparing the prediabetes group and the diabetes group, the differences in T1 relaxation time, mean secretion grade and frequency of the pancreatic secretory inflow were not significant (*p* = 0.406, *p* = 0.904 and, *p* = 1.000, respectively).

**Table 1:** Comparison of the MR measurements among the three groups

Parameter	Normal group (*n* = 12)	Prediabetes group (*n* = 15)	Diabetes group (*n* = 16)	*P*
T1 relaxation time of pancreas (msec)	803 (750–910)	934 (850–1024)	968 (851–1191)	0.034
Frequency of pancreatic juice secretion observed	12 (6.75–16)	2 (0.5–13.5)	2 (0.75–9.5)	0.093
Mean secretion grade	0.8 (0.34–1.25)	0.1 (0.025–0.775)	0.1 (0.075–0.725)	0.095

Data are median with 25th and 75th percentiles in parentheses

In the comparison between the normal group and the group with elevated HbA1c (i.e. prediabetes and diabetes, *n* = 31) (Table 2), the frequency of the pancreatic secretory inflow was significantly lower in the elevated HbA1c group than in the normal group (median, 2 [interquartile range 0.5–12] times vs. median, 12 [interquartile range 6.75–16] times, *p* = 0.029). In addition, the mean secretion grade was also significantly lower in the elevated HbA1c group than in the normal group (median, 0.1 [interquartile range 0.025–0.75] vs. median, 0.8 [interquartile range 0.34–1.25], *p* = 0.030), indicating that the pancreatic juice secretion was significantly decreased in the patients with elevated HbA1c.

**Table 2:** Comparison of the MR measurements between the normal HbA1c group and the elevated HbA1c group

Parameter	Normal group (*n* = 12)	Elevated HbA1c group (*n* = 31)	*P*
T1 relaxation time of pancreas (msec)	803 (750–910)	944 (850–1085)	0.013
Frequency of pancreatic juice secretion observed	12 (6.75–16)	2 (0.5–12)	0.029
Mean secretion grade	0.8 (0.34–1.25)	0.1 (0.025–0.75)	0.030

Data are median with 25th and 75th percentiles in parentheses

## Discussion

Our findings showed that both the mean secretion grade and frequency of the pancreatic secretory inflow in cine-dynamic MRCP with a spatially selective IR pulse had a significant negative correlation with the HbA1c values. In addition, both the mean secretion grade and frequency of the pancreatic secretory inflow in the elevated HbA1c group were significantly lower than those in the normal group. A previous study demonstrated that the cut-off value of 0.7 for the mean secretion grade in cine-dynamic MRCP was the criterion of pancreatic exocrine dysfunction [6]. This indicates that the patients in the elevated HbA1c group whose median mean secretion grade was 0.1 had an impaired pancreatic exocrine function. Therefore, our findings suggest that the pancreatic exocrine function might be reduced in patients with an impaired pancreatic endocrine function.

In this study, the T1 relaxation time of the pancreatic parenchyma showed a significant positive correlation with the HbA1c values, in accordance with a previous study [11]. In addition, we also found that both the mean secretion grade and frequency of the pancreatic secretory inflow showed a significant negative correlation with the T1 relaxation time of the pancreatic parenchyma. These findings suggested that the reduced pancreatic exocrine function was interrelated with the decline in the pancreatic endocrine function, and indicated that a combination of cine-dynamic MRCP with a spatially selective IR pulse and T1 mapping might have potential for estimating both the pancreatic endocrine and exocrine function noninvasively cooperating with physiological pancreatic function tests.

In this study, both the mean secretion grade and frequency of the pancreatic secretory inflow tended to be lower in the patients with prediabetes and DM than in those with a normal pancreatic endocrine function. This suggests that the pancreatic exocrine function might already be decreased in patients with prediabetes. Previous studies on the pancreas histology of diabetic patients showed many histopathological findings and structural changes, such as atrophy with decreased acinar and beta-cell, chronic inflammation, fibrosis due to microvascular complications, arteriosclerosis, nerve fiber loss and morphological changes in the pancreatic duct [12–14], and these changes can contribute to the development of pancreatic exocrine insufficiency. Thus far, little has been reported concerning the pancreatic exocrine function in patients with prediabetes. However, a decreased pancreatic beta-cell mass, microvascular damage and neuropathy have been also reported in patients with prediabetes [15, 16]. Therefore, pancreatic exocrine insufficiency can be seen in patients with prediabetes and early DM who have mild pancreatic endocrine dysfunction. In addition, our results suggest that cine-dynamic MRCP with a spatially selective IR pulse may be useful in the early diagnosis of pancreatic exocrine dysfunction in patients with mild pancreatic endocrine insufficiency, so early therapeutic intervention for pancreatic exocrine dysfunction is clinically desirable for patients with prediabetes or DM.

While we expected that the pancreatic exocrine function would be reduced with the progression of pancreatic endocrine dysfunction, no significant differences in the mean secretion grade or frequency of the pancreatic secretory inflow were observed between the patients with prediabetes and those with DM. In a cohort study of 667 patients with DM, Larger et al. [17] found no correlation between pancreatic exocrine insufficiency and the duration of DM. A long-term follow-up study suggested that mild to moderate pancreatic exocrine insufficiency had been present since the onset of DM and had not progressed [18]. These data suggest that the pancreatic exocrine function might not be significantly decreased during the transition from prediabetes to diabetes.

Several limitations associated with the present study warrant mention. First, because this study was retrospective and the number of patients was relatively small, there may have been potential selection bias. Second, the evaluation of the pancreatic secretory inflow within the area with a spatially selective IR pulse in cine-dynamic MRCP images may be subjective. However, the interobserver agreement for the assessment of the inflow of the pancreatic juice with cine-dynamic MRCP was excellent. Third, study population included patients with branch-duct intraductal papillary mucinous neoplasms which could increase the viscosity of pancreatic juice. However, the size of IPMNs was relatively small, and no patients showed the dilated pancreatic duct suggesting stagnant pancreatic flow. Therefore, it is less likely to affect the flow dynamics of pancreatic juice in these patients with IPMNs, and we believe that the validity of this study will not be compromised. Finally, we classified patients into three groups according to the HbA1c values. Although the measurement of HbA1c is adequate for the diagnosis of diabetes as well as the measurement of fasting plasma glucose and 2-h plasma glucose value during a 75-g oral glucose tolerance test, HbA1c is an indirectly measured value of the average blood glucose levels and may be influenced by a number of factors, such as age, race and presence of anemia [19]. Therefore, further clinical studies using other pancreatic endocrine function tests, including the measurement of C-peptide and glucagon levels, will be needed to validate our results.

In conclusion, the pancreatic exocrine function estimated by cine-dynamic MRCP with a spatially selective IR pulse was significantly decreased in patients with prediabetes and DM, suggesting the interrelation between the pancreatic exocrine and endocrine functions. Cine-dynamic MRCP with a spatially selective IR pulse may contribute to the diagnosis of pancreatic exocrine insufficiency in patients with pancreatic endocrine insufficiency.
